# Correlation between age and the sciatic nerve diameter in the first 2 years of life: A high‐resolution ultrasound study

**DOI:** 10.1002/brb3.2944

**Published:** 2023-03-22

**Authors:** Carole Jenny, Jacoba van der Linde, Thomas Hundsberger, Philip J. Broser

**Affiliations:** ^1^ Medical Faculty University of Basel Switzerland; ^2^ Department of Neurology Cantonal Hospital St. Gallen Switzerland; ^3^ Department of Neuropaediatrics Children's Hospital of Eastern Switzerland St. Gallen Switzerland

**Keywords:** high‐resolution ultrasound imaging, maturation of the peripheral nervous system

## Abstract

**Aim:**

To investigate the maturation of the peripheral nervous system by analyzing the cross‐sectional area of the sciatic nerve during the first 2 years of life.

**Methods:**

The sciatic nerve was examined by high‐resolution ultrasound imaging in 52 children aged 0 days to 10 years, 45 of whom were younger than 2 years. The correlation between the cross‐sectional area of the nerve and the age was statistically tested. A logarithmic regression analysis was performed to develop a logarithmic growth model of the cross‐sectional area.

**Results:**

There is a highly significant correlation between the age and the cross‐sectional area of the sciatic nerve. The growth rate can well be described by a logarithmic model.

**Interpretation:**

Based on the literature on the maturation of the median nerve and nerve roots and the findings of the present study, we conclude that both the proximal and the distal parts of the nerves of the peripheral nervous system increase simultaneously.

**What this paper adds:**

Normative values for the size of the sciatic nerve in children.

## Graphical abstract

The sciatic nerve was examined by high‐resolution ultrasound imaging in children younger than two years. There is a highly significant correlation between the age and the cross‐sectional area of the sciatic nerve. The growth rate can well be described by a logarithmic model.

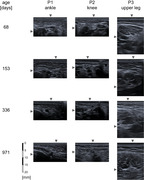

## 1 INTRODUCTION

It is well known that an increase in nerve conduction velocity is an essential component of the maturation of the peripheral nervous system in children (Raimbault, 1988). Until the development of high‐resolution ultrasound imaging, most knowledge about peripheral nervous system maturation, such as myelination and growth of the nerve sheath, was obtained by in vitro studies or in animal experiments (Kaplan et al., 2009). There are some available data on the size of the cross‐sectional area of the sciatic nerve in adults, measured by high‐resolution ultrasound (Bae & An, 2022; Cartwright et al., 2013; Cartwright et al., 2008; Druzhinin et al., 2019; Niu et al., 2021; Schubert et al., 2020; Singh et al., 2021). However, there are only a few studies on children, with most of these focusing on children older than 2 years (Bae & An, 2022; Cartwright et al., 2013; Cartwright et al., 2008; Druzhinin et al., 2019; Niu et al., 2021; Schubert et al., 2020; Singh et al., 2021). Our group has previously shown that the size of the median nerve and the nerve roots C5 and C6, change significantly during the first years of life (Jenny et al., 2020; Van Der Linde et al., 2022). Presumably, this is a sign of maturation, as the nerve conduction velocity increases in a same logarithmic manner (García‐García & Calleja‐Fernández, 2004; Parano et al., 1993). However, it is unclear whether the proximal parts of the nerve and its distal parts mature in the same way and whether the nerves of the lower extremity follow the same growth patterns as the nerves of the upper extremity (Van Der Linde et al., 2022).

In this study, our aim was to extend this knowledge to the sciatic nerve and to add standard values for the size of the nerve. With this aim in mind, we measured the size of the cross‐sectional area of the sciatic and the tibial nerve using high‐resolution ultrasound in children aged 0–2 years (main group) and children up to 10 years (comparison group to follow existing studies) (Schubert et al., 2020).

## 2 MATERIALS AND METHODS

This was a prospective cross‐sectional study, which was approved by the ethics committee of St. Gallen (EKOS approval no: EKSG 19/166). All the participants’ legal representatives were fully informed about the study, any potential risks and the rights of the participants prior to the study, and their written consent was obtained.

The study population consisted of 56 children who were hospitalized in the Children's Hospital of Eastern Switzerland for various reasons between September 2020 and February 2022. Most of these children (39) already participated in a prior study by van der Linde et al. (2022). The majority of the participants were younger than 2 years (*n* = 45). Eleven children aged older than 2 years were included for comparison with existing data (Bae & An, 2022; Cartwright et al., 2013; Cartwright et al., 2008; Druzhinin et al., 2019; Niu et al., 2021; Schubert et al., 2020; Singh et al., 2021). The target study size was 50. Exclusion criteria were preexisting neurological disorders or chronic diseases, premature birth or a family history of neurological disorders. Imaging was performed just before discharge from the hospital. Therefore the participants were in relatively good health.

To optimize the reproducibility of the study, a strict examination and measurement protocol was followed. All measurements were performed using the same Canon Aplio i800 machine (Canon Medical Systems, Tokyo, Japan) with a i18LX5 probe (maximum scanning frequency of 18 MHz), with the same settings established by Jenny et al. (2020) and Van Der Linde et al. (2022). According to the protocol, the child was in a prone position at the beginning of the examination. However, this position was not always maintained during the whole examination because of the natural movements of the child, the interaction with the parents or the general non‐compliance of the child.

**FIGURE 1:**
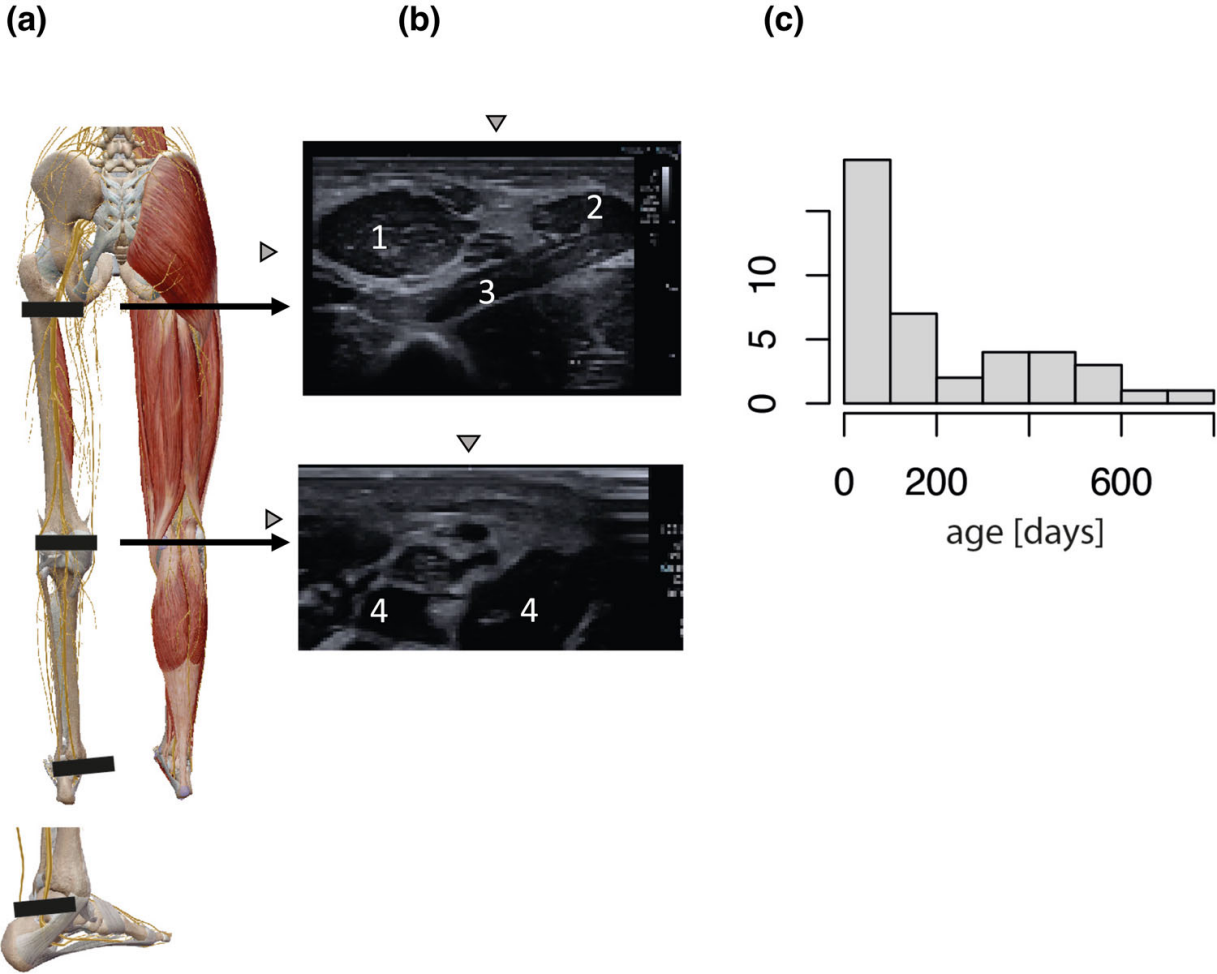
(A) Placement of the ultrasound probe on the leg to scan the three different positions (black bars). (B) Anatomical structures surrounding the nerve. 1: Musculus gluteus maximus 2: Musculus biceps femoris 3: Musculus quadratus femoris; 4: medial and lateral head of the gastrocnemius muscle. (C) Age distribution of the main study group in 100‐day intervals.

The sciatic nerve was identified with the help of anatomical landmarks, as well as the typical sonographic appearance of a peripheral nerve (Gruber et al., 2018). The sciatic, respectively the tibial nerve was visualized at three positions (Figure 1). Position 1 was set just dorsal to the medial malleolus where the tibial nerve is close to the posterior tibial artery (Figure 1, panel A). Position 2 was located at the popliteal area where the tibial nerve is bedded in connective tissue. Position 3 was set at the gluteal region just below the greater sciatic foramen where the sciatic nerve runs between the lateral rotators of the hip and the gluteus maximus muscle (Elsevier, 2022).

If possible, the nerves in both legs were examined. To exploit one of the greatest advantages of sonography, its dynamics, a short video recording was taken at each of the three predetermined positions, and after the examination, a still image was selected from the video recordings.

Using the freehand tracing tool of the Canon Aplio i800, the cross‐sectional area of the nerve was calculated based on the circumference of the nerve's outline (Figure S2). This procedure was established and validated in the previous study of Jenny et al. (2020). The cross‐sectional area was then provided in square millimeters and the circumference in millimeters (Jenny et al., 2020). After the data were obtained for every subject and every position, additional patient information, such as age, sex, height, and weight, was added to the study database. The data were stored and encrypted.

Using the plotting tool of R (R Core Team, 2018) the obtained values for the cross‐sectional area were plotted against the corresponding age of each participant. The scatter plots were divided into the two study groups: the main study group and the comparison group (Figures 3, 4, 5).

To test for statistically significant differences in the data obtained from the left and right leg, as well as for differences in the data in males and females, a Mann–Whitney *U* test was used. Kendall's correlation test was used to investigate correlations between age, weight and nerve cross‐sectional area (Hollander et al., 1973). The visual representation of the scatterplot suggested a correlation of age and the cross‐sectional area and the curve seems to follow a logarithmic pattern (Figures 3, 4, 5). This was tested with a logarithmic regression analysis (Wilkinson & Rogers, 1973).

As this examination method is examiner‐dependent, interrater variability was prevented by having only one of the authors conduct the examinations and the following measurements. The intrarater variability for this author was tested and calculated before (Jenny et al., 2020).

## 3 RESULTS

### 3.1 Study population

In total, 56 children were screened. Of these, 52 were included in the final study. Most of these children (39) already participated in a prior study by van der Linde et al. (2022). One child was excluded because of premature birth, one was excluded because of Hirschsprung's disease, one was excluded because of low imaging quality, and one was excluded because of withdrawal from the study. Panel C of Figure 1 shows the age distribution of the final main group and the comparison group.

### 3.2 Imaging of the sciatic nerve

**FIGURE 2:**
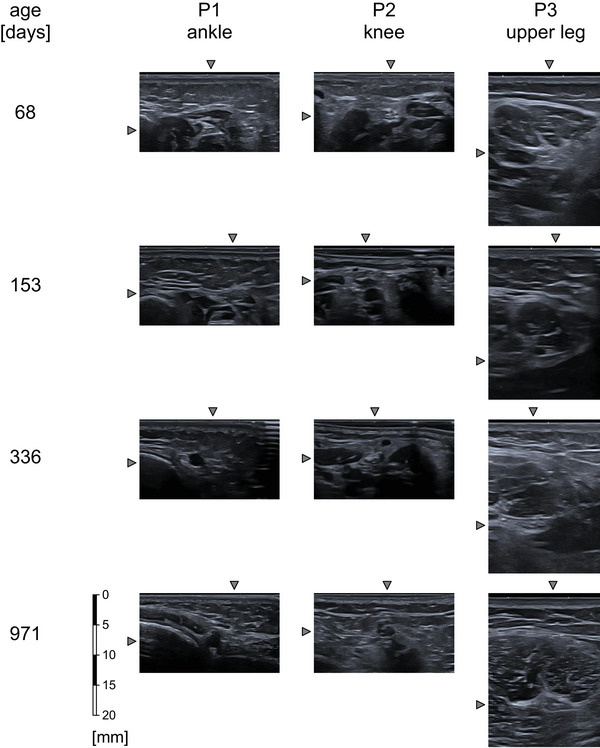
Typical ultrasound images of the sciatic nerve in the three different positions at various ages. The arrows next to the images point to the nerve. The 20 mm scale at the bottom left of the figure applies to all images.

Panel B of Figure 1 shows typical ultrasound images of the sciatic and the tibial nerve and its anatomical surroundings. Figure 2 illustrates the absolute increase in the size of the cross‐sectional area of the nerve with increasing age at the three predefined positions.

### 3.3 Cross‐sectional area

To provide a quantitative measure of the increase in nerve size, the cross‐sectional area was measured in square millimeters in all the participants. The measurements and additional patient information of the whole study group are shown in Table 1.

**TABLE 1:** Demographics and measurements of the cross‐sectional area of the sciatic nerve

N.ischadicus data									
Demographics				Area (mm^2^)					
Age (day)	Weight (kg)	Length (cm)	Sex	R1F	R2F	R3F	L1F	L2F	L3F
1	3.9	53	M	2.23	3.83	10.08	3.23	3.70	7.60
5	3.5	52	M	2.92	4.78	5.53	3.07	4.33	7.38
9	3.3	48	M	1.99	3.61	7.80	2.48	3.51	7.84
10	3.0	50	F	2.26	6.37	7.53	1.79	5.23	5.42
14	3.7	51	F	3.51	4.21	7.58	3.25	3.15	8.45
16	4.7	55	F	2.00	3.24	7.40	2.01	3.40	5.42
25	4.4	53	M	2.32	3.18	4.20	2.69	2.88	4.96
38	3.7	50	F	2.05	3.01	4.79	2.22	2.74	6.17
39	5.5	57	M	3.18	3.85	7.55	3.76	4.17	7.98
40	3.8	56	F	2.62	3.31	5.59	2.34	3.11	5.85
41	4.2	52	F	1.94	3.19	6.40	2.17	3.27	6.53
53	5.2	54	F	3.03	5.11	7.68	3.41	4.98	7.12
55	3.8	56	M	4.44	4.72	8.58	4.54	3.64	7.80
60	5.4	60	M	1.91	3.46	6.11	1.96	3.33	10.40
63	4.1	57	M	3.56	3.97	5.73	3.67	3.88	10.71
68	4.8	57	F	2.42	3.28	7.90	2.35	4.11	11.30
80	6.2	62	M	2.94	6.52	9.88	3.25	4.21	10.12
82	5.6	64	M	3.04	3.61	8.70	3.87	3.86	8.82
93	—	—	F	4.04	4.18	7.56	3.08	4.60	9.89
107	6.5	61	M	3.43	3.85	10.10	3.46	4.10	11.10
123	3.8	49	F	3.10	3.38	7.78	3.67	4.76	6.15
135	6.2	62	F	3.80	4.13	6.16	3.35	4.41	6.58
141	8.1	68	F	3.12	4.06	8.90	3.10	3.80	10.30
145	8.4	64	M	—	—	—	3.20	4.16	11.40
153	7.4	67	M	4.06	4.53	10.90	4.04	4.66	10.80
187	7.4	73	M	3.03	6.30	9.48	4.96	4.71	9.28
227	9.8	72	M	3.30	4.70	10.36	3.28	4.21	10.01
257	8.6	75	M	2.68	5.43	10.99	3.39	—	9.62
306	7.4	70	F	4.31	5.05	10.92	5.16	4.60	9.32
322	7.1	69	F	—	3.61	—	—	—	—
325	8.8	79	M	3.64	4.18	9.67	3.90	4.12	9.82
336	10.5	74	M	3.22	3.93	9.10	3.42	3.91	10.60
401	11.0	77	M	4.78	5.67	11.20	4.58	5.86	12.10
417	11.6	79	M	—	4.91	10.00	3.36	5.20	9.84
473	9.9	74	M	3.01	5.64	17.20	3.82	9.50	17.30
484	8.3	78	F	3.61	7.37	11.99	3.63	6.62	9.62
584	10.4	80	M	3.21	4.57	11.70	—	5.39	—
590	9.9	—	M	4.91	5.10	10.00	5.18	5.06	10.20
598	12.7	85	F	3.32	4.99	13.20	3.55	4.62	12.00
670	12.0	86	M	4.67	12.90	13.60	4.47	12.29	17.70
711	12.5	—	M	4.53	6.14	—	4.16	6.17	—
750	12.2	85	F	5.37	9.58	—	5.45	—	—
781	13.0	88	F	5.17	5.99	—	4.55	6.56	—
960	14.5	—	M	5.11	—	—	4.65	—	—
1039	12.8	—	M	3.80	4.73	11.40	3.92	4.73	12.40
1205	15.0	—	F	4.19	4.93	11.80	4.81	4.56	10.00
1405	14.5	100	M	3.48	7.94	—	3.06	7.10	—
1623	17.0	—	F	—	—	—	5.55	8.46	—
2131	7.6	65	M	7.40	8.40	16.30	—	—	—
2344	22.0	115	M	4.55	6.95	—	4.87	7.11	—
3123	26.0	—	M	7.31	9.54	—	5.86	11.48	—
3753	32.9	144	M	4.21	13.30	—	5.64	13.60	—

The Mann–Whitney *U* test did not show any significant differences between the nerve of the left and right leg (location 1: mean difference [R‐L] = 0.15 mm^2^, location 2: mean difference [R‐L] = 0.02 mm^2^, location 3: mean difference [R‐L] = −0.33 mm^2^, *p* > 0.3 for all). Further, a matched pairs test did not show any sex related differences.

**FIGURE 3:**
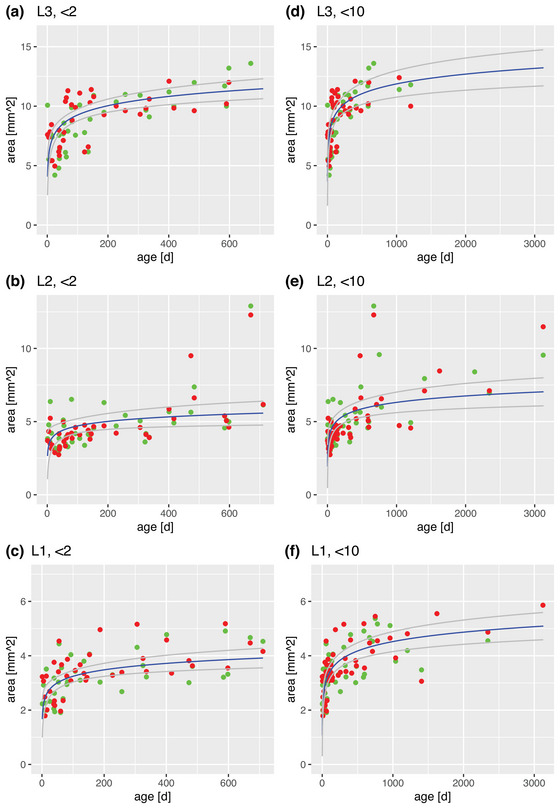
Scatterplots relating age and cross‐sectional area (green dots: right leg, red dots: left leg). Panels A–C illustrate the main study group; panels D–F illustrate the comparison group; blue line: logarithmic regression curve with a 5% and 95% confidence‐interval (gray lines).

Figure 3 shows the cross‐sectional area plotted against the age of the subject at the time of the measurement. In accordance with the visual inspections, which suggested an increase in the cross‐sectional area with age, Kendall's correlation test revealed a highly significant correlation between age and the cross‐sectional area at all three locations (*p* value < 10^−5^: location 1: tau = 0.56, *p* value = 2.333e‐08; location 2: tau = 0.4895614, *p* value = 9.662e‐07; location 3: tau = 0.63, *p* value = 6.35e‐09.)

### 3.4 Logarithmic regression between age and the cross‐sectional area

The scatterplot in Figure 3 shows that the increase in cross‐sectional area is greatest during early life and then slows down. This is a typical behavior of a logarithmic model. Therefore, the hypothesis was tested if the increase is governed by a logarithmic model:

Cross‐sectional area = *b* + *a* × log (age [days])—(with *a*: the intersection and *b*: the slope).

For each location, the model was tested, and a regression analysis was performed. The results revealed highly significant values (Table 2).

**TABLE 2:** Statistics of logarithmic model

Loc.	*a*	*b*	*R* ^2^	Std. error	*p* Value
1	1.66732	0.34239	0.38242	0.07252	.00004
2	2.65400	0.44539	0.15584	0.16816	.01171
3	4.07195	1.12625	0.37692	0.16833	.00000

### 3.5 Weight and length

**FIGURE 4:**
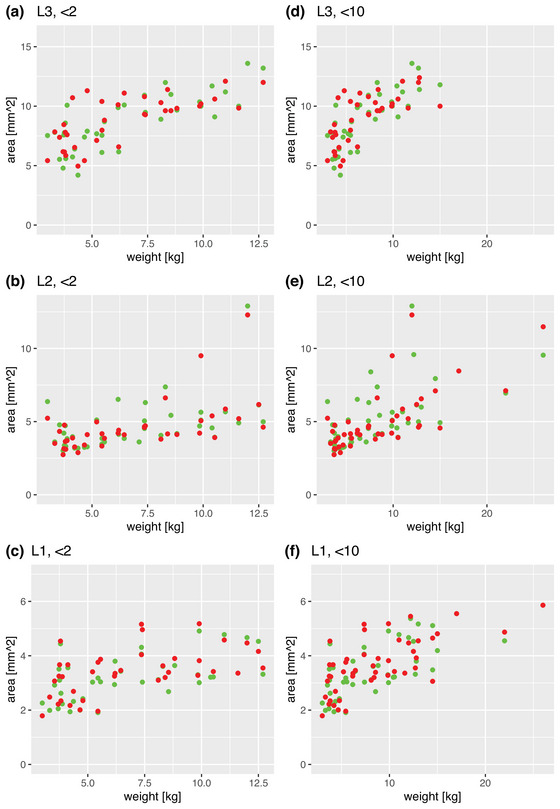
Scatterplots relating weight and cross‐sectional area (green dots: right leg, red dots: left leg). Panels A–C illustrate the main study group; panels D–F illustrate the comparison group.

**FIGURE 5:**
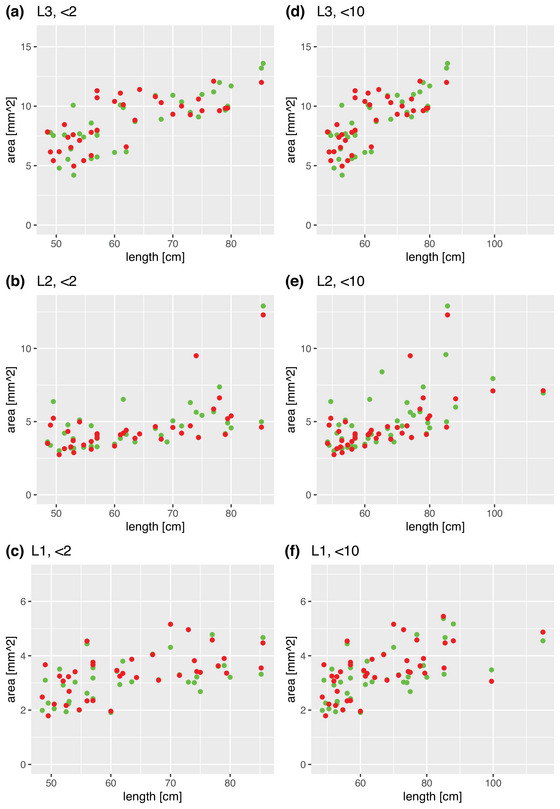
Scatterplots relating weight and cross‐sectional area (green dots: right leg, red dots: left leg). Panels A–C illustrate the main study group; panels D–F illustrate the comparison group.

Given that also weight and length changes with age in a semi‐logarithmic manner, it was expected to see a linear relationship between cross‐sectional area and weight and length. As demonstrated in Figures 4 and 5, the data show this type of relation.

### 3.6 Normative data

To increase the applicability of the data to daily medical practice, standard values were generated for children under 2 years of age (Figure S1).

## 4 DISCUSSION

As already found by Jenny et al. (2020) and Van Der Linde et al. (2022), the size of the median nerve and nerve roots C5 and C6 is correlated with the age of a child. The aim of this study was to determine whether the sciatic nerve, a nerve of the lower extremity, follows the same maturation dynamics as described previously for the median nerve and the nerve roots C5 und C6. This study revealed a consistent increase in the cross‐sectional area of the nerve, with no difference for sex and side. Based on our findings, the growth rate is largest during the first 24 months of life to then slow down over time. Like the median nerve (Jenny et al., 2020) and nerve roots (Van Der Linde et al., 2022), the growth rate of the sciatic nerve follows a similar logarithmic pattern. By comparison with existing data for adults (Schubert et al., 2020), we estimate that 70% of the final size of the cross‐sectional area of the sciatic nerve is reached at about 2 years of age.

From the neurophysiological data available (Pitt, 2017; Raimbault, 1988), we know that the nerve conduction speed changes with a similar pattern for the peroneal and tibial nerve like the increase in the cross‐sectional area. Considering the similarity in the structural and the functional maturation of the median and the sciatic nerves, we can conclude that the maturation of the peripheral nervous system is similar in the proximal and the distal parts of the nerves.

The limitations of this study were the small sample size and the age distribution, with many children younger than 6 months screened but fewer children older than 6 months (Figure 1, Panel C).

A longitudinal study design could illustrate growth better. As noted previously by Beekman and Visser (2004), there are certain circumstances that make it more difficult to image the nerves of the lower extremity using high‐resolution sonography in comparison to the median nerve or the nerves of the upper extremity in general.

As a further limitation this study focused only on the size of the cross‐sectional area of the complete nerve. As already shown by Ricci et al. (2022), it is also possible to differentiate structures within the nerve, such as the fascicular plexus with the help of high‐resolution ultrasound. It would be interesting in a future study to measure these internal structures and to analyze the ratio between neural and non‐neural tissue during the maturation of the peripheral nerve.

Current understanding of the maturation of the peripheral nervous system suggests that the increase in size of the cross‐sectional area of a nerve is due not to an increase in the number or size of the axons but an increase in size of the myelin sheaths (Niebrój‐Dobosz et al., 1980). Therefore, the assumption that the increase in size and the increase in nerve conduction velocity correlate is plausible (Broser & Lütschg, 2020).

To gain more insight, this problem should be further addressed in a simultaneous morphological and functional study by electroneurographically investigating the nerve conduction velocity and sonographically measuring the cross‐sectional area of a nerve in the same subject in a longitudinal study.

## CONFLICT OF INTEREST STATEMENT

The authors declare no conflicts of interest.

### PEER REVIEW

The peer review history for this article is available at https://publons.com/publon/10.1002/brb3.2944.

## Supporting information

Figure S1. Normative values for the size of the cross‐sectional area of the sciatic nerve divided into age groups of 120 days.

Figure S2. Typical ultrasound images of the sciatic nerve in the three different positions to illustrate how the outline of the nerve was traced and measured. The arrows next to the images point to the nerve.

## Data Availability

All relevant data for this study is included in the main document.
