# Supplementary figures and images for: Correlation between age and the sciatic nerve diameter in the first 2 years of life: A high‐resolution ultrasound study
Source: Brain Behav. 2023 Mar 22;13(4):e2944. doi: 10.1002/brb3.2944 (PMC10097064; doi:10.1002/brb3.2944)

**A** Pos 3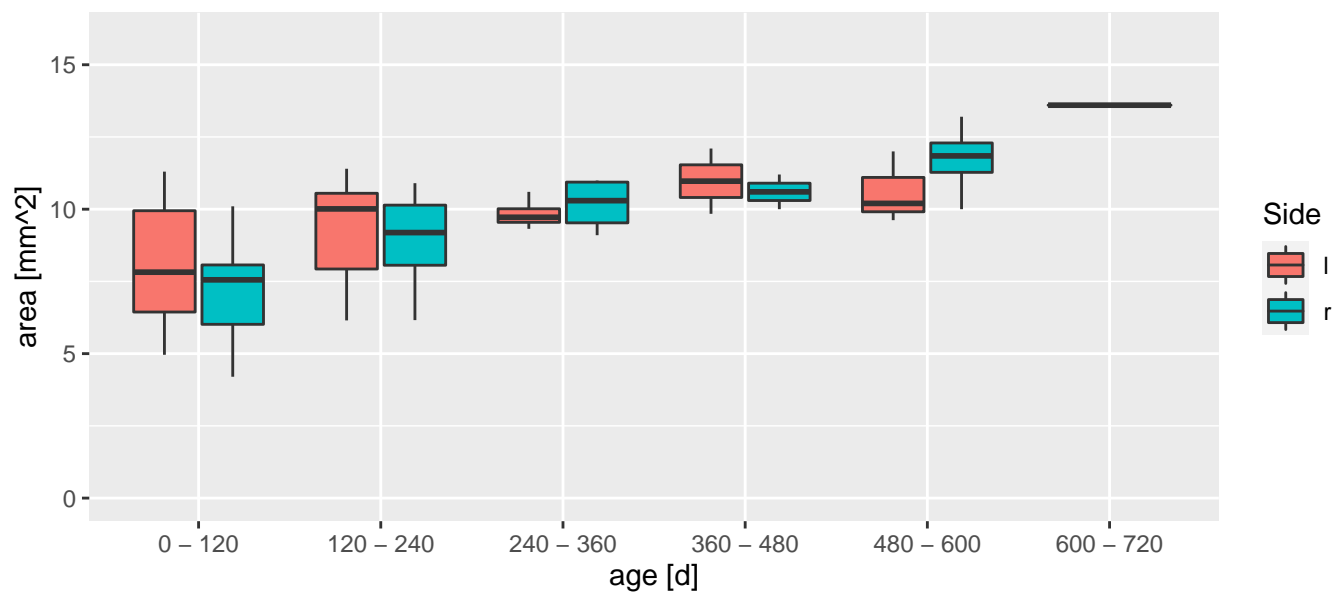**B** Pos 2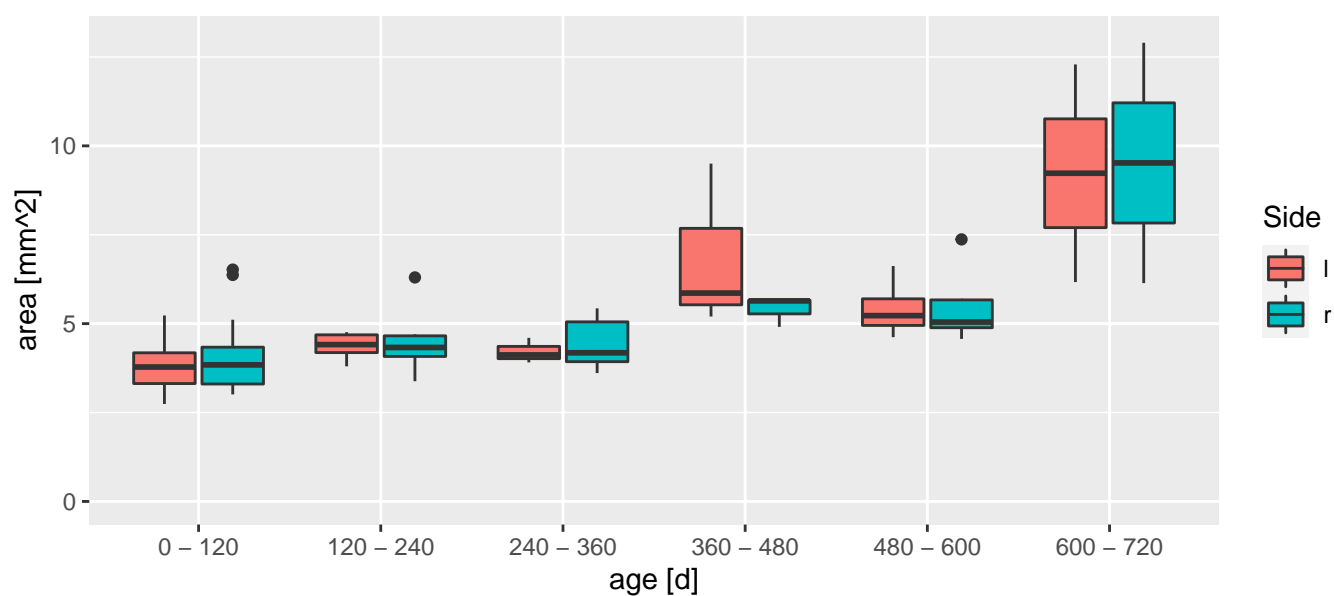**C** Pos 1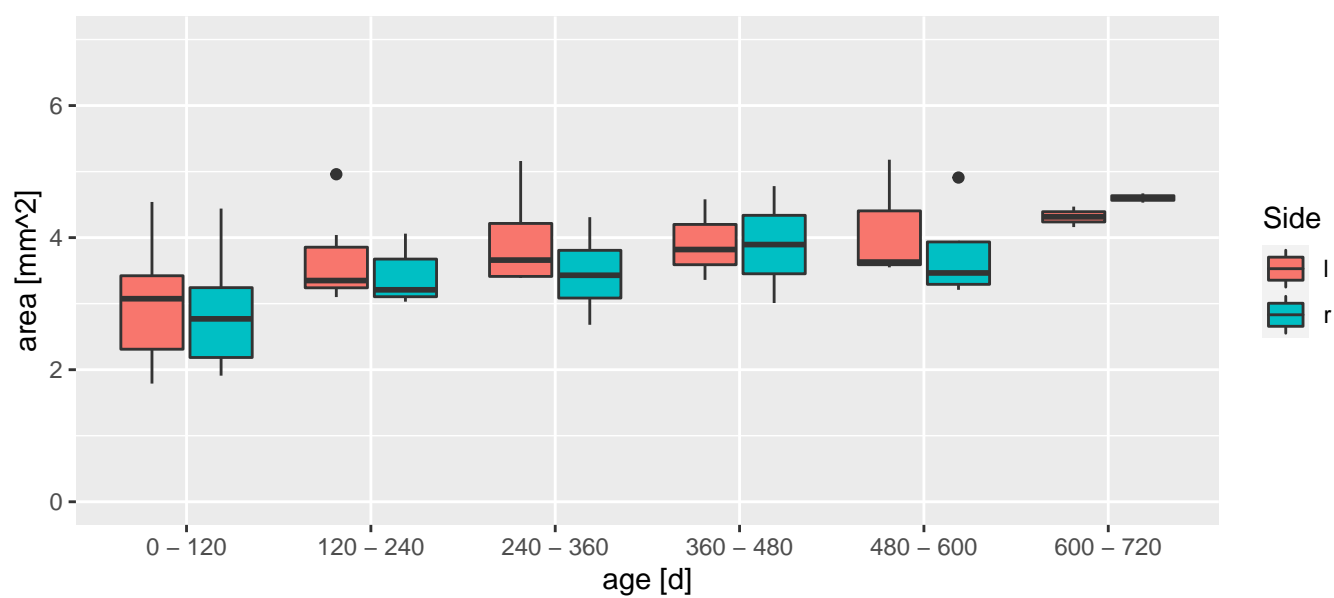

Supplement: Supplementary file 1 — Figure S1. Normative values for the size of the cross‐sectional area of the sciatic nerve divided into age groups of 120 days. [file BRB3-13-e2944-s001.pdf]

Pos 1

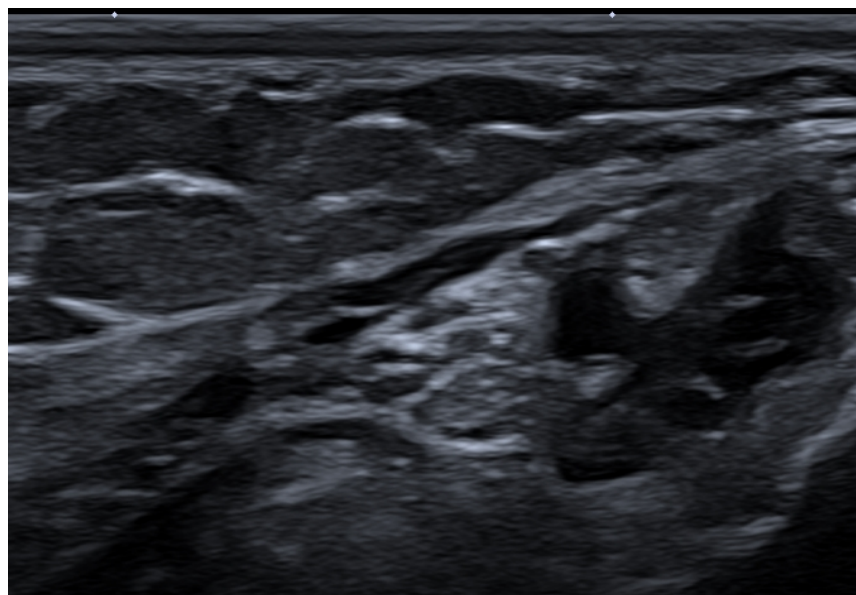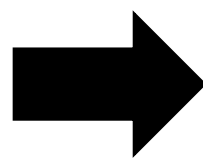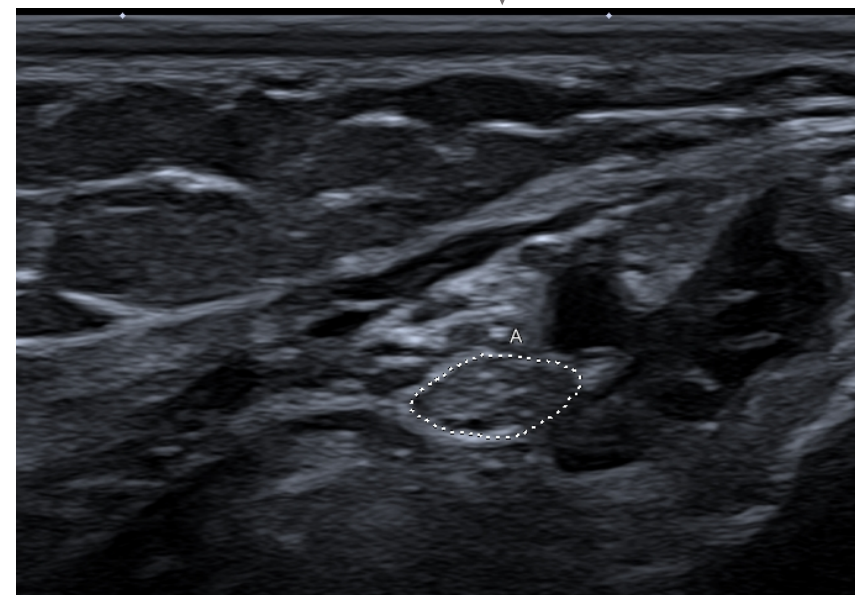

Pos 2

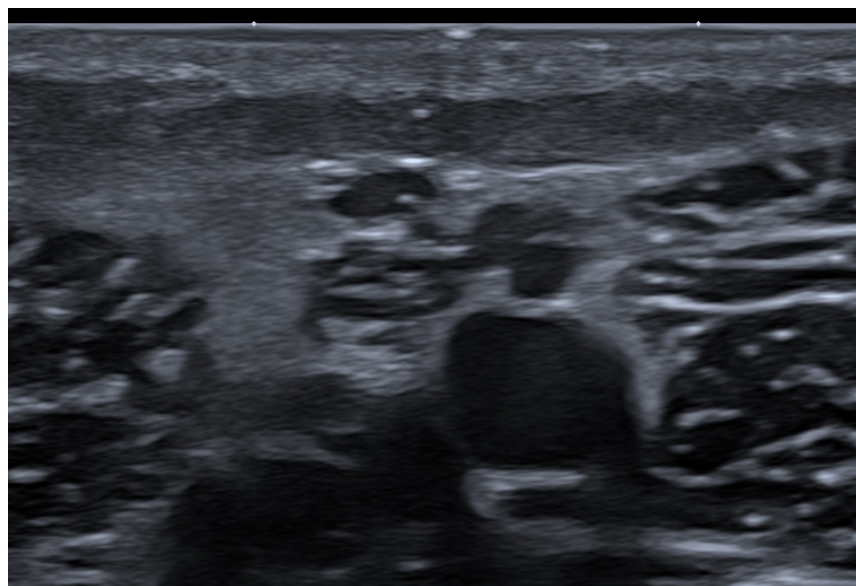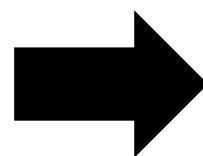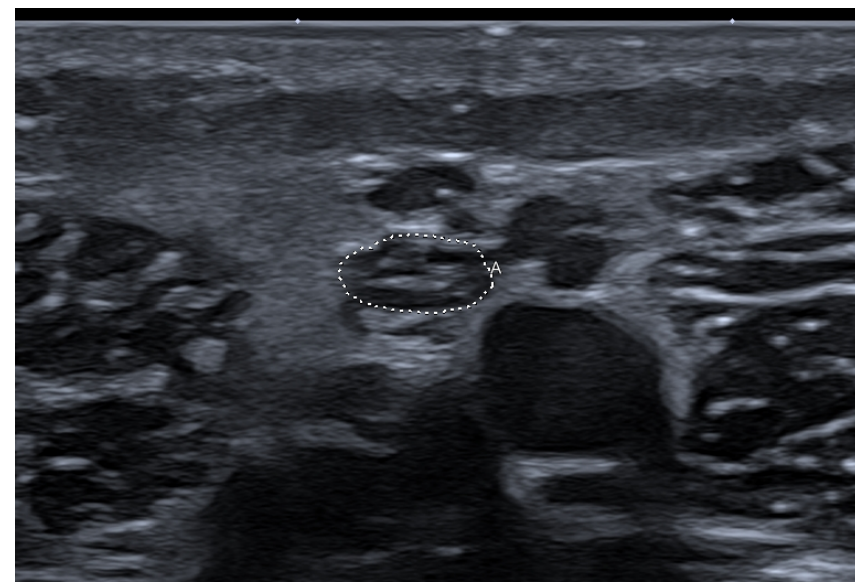

Pos 3

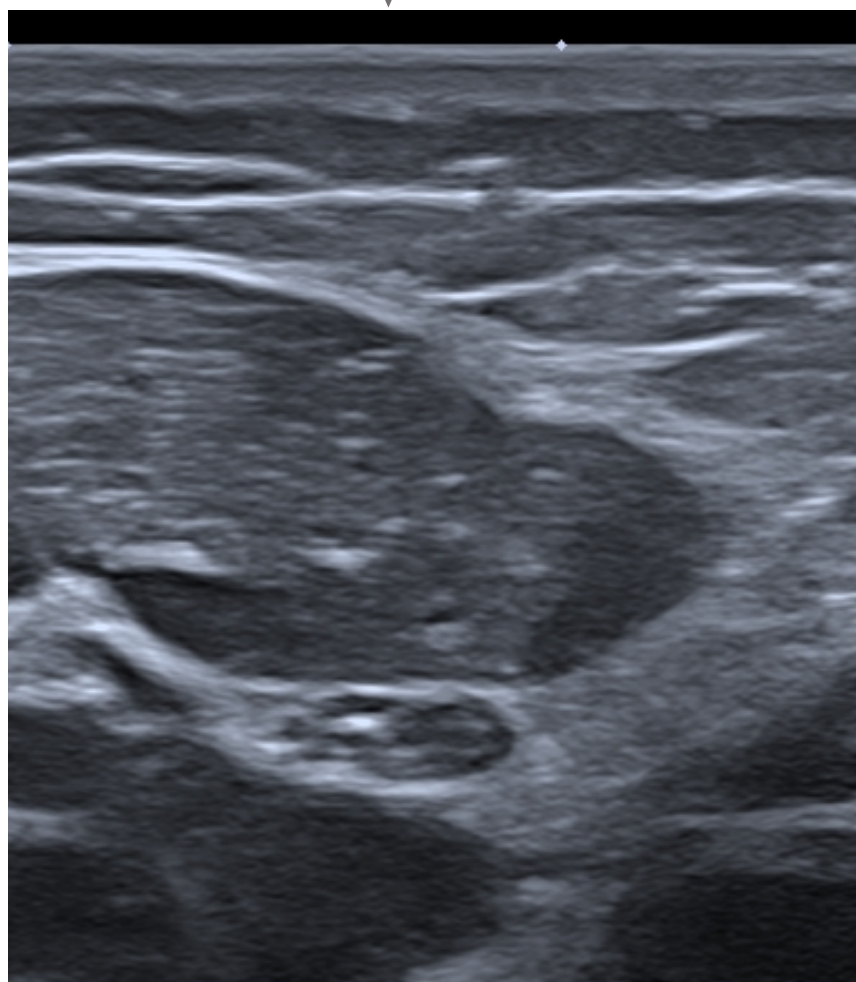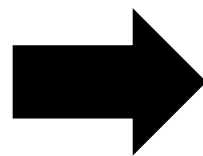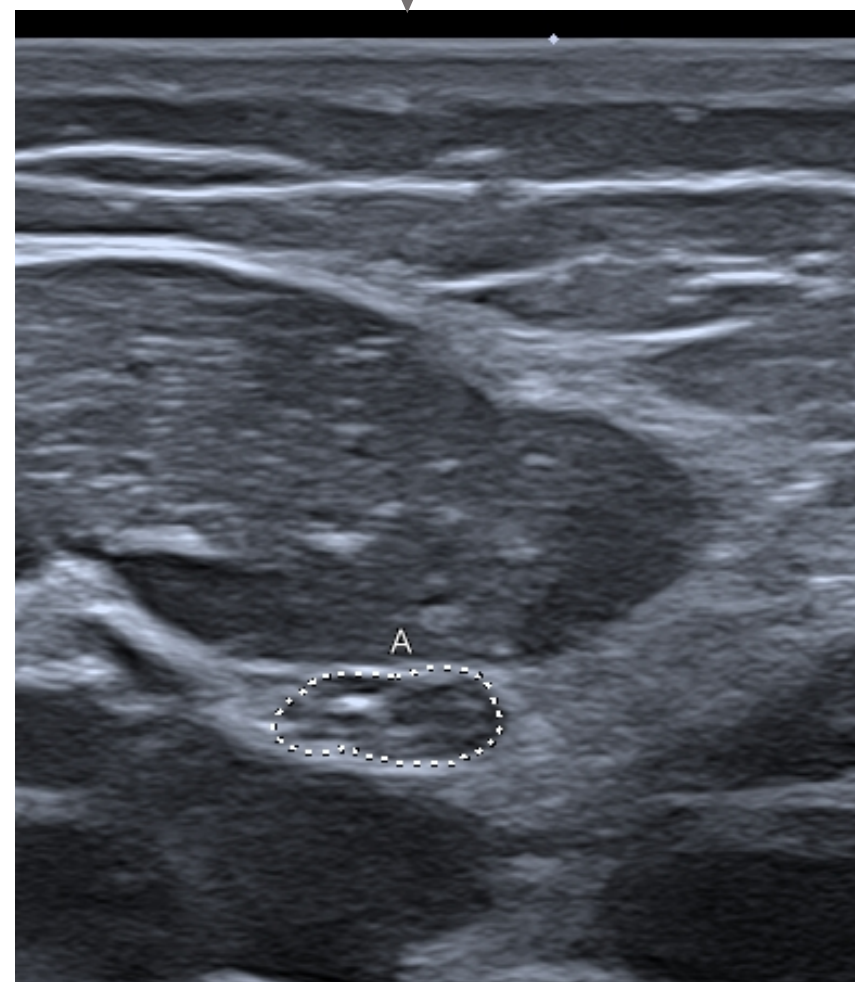

Supplement: Supplementary file 2 — Figure S2. Typical ultrasound images of the sciatic nerve in the three different positions to illustrate how the outline of the nerve was traced and measured. The arrows next to the images point to the nerve. [file BRB3-13-e2944-s002.pdf]
